# Mechanism of the Interaction between the Intrinsically Disordered C-Terminus of the Pro-Apoptotic ARTS Protein and the Bir3 Domain of XIAP

**DOI:** 10.1371/journal.pone.0024655

**Published:** 2011-09-20

**Authors:** Tali H. Reingewertz, Deborah E. Shalev, Shahar Sukenik, Ofrah Blatt, Shahar Rotem-Bamberger, Mario Lebendiker, Sarit Larisch, Assaf Friedler

**Affiliations:** 1 Institute of Chemistry, Hebrew University of Jerusalem, Safra Campus, Givat Ram, Jerusalem, Israel; 2 The Wolfson Centre for Applied Structural Biology, Hebrew University of Jerusalem, Safra Campus, Givat Ram, Jerusalem, Israel; 3 Cell Death Research Laboratory, Department of Biology, Faculty of Natural Sciences, University of Haifa, Mount Carmel, Haifa, Israel; University of South Florida College of Medicine, United States of America

## Abstract

ARTS (Sept4_i2) is a mitochondrial pro-apoptotic protein that functions as a tumor suppressor. Its expression is significantly reduced in leukemia and lymphoma patients. ARTS binds and inhibits XIAP (X-linked Inhibitor of Apoptosis protein) by interacting with its Bir3 domain. ARTS promotes degradation of XIAP through the proteasome pathway. By doing so, ARTS removes XIAP inhibition of caspases and enables apoptosis to proceed. ARTS contains 27 unique residues in its C-terminal domain (CTD, residues 248–274) which are important for XIAP binding. Here we characterized the molecular details of this interaction. Biophysical and computational methods were used to show that the ARTS CTD is intrinsically disordered under physiological conditions. Direct binding of ARTS CTD to Bir3 was demonstrated using NMR and fluorescence spectroscopy. The Bir3 interacting region in ARTS CTD was mapped to ARTS residues 266–274, which are the nine C-terminal residues in the protein. Alanine scan of ARTS 266–274 showed the importance of several residues for Bir3 binding, with His268 and Cys273 contributing the most. Adding a reducing agent prevented binding to Bir3. A dimer of ARTS 266–274 formed by oxidation of the Cys residues into a disulfide bond bound with similar affinity and was probably required for the interaction with Bir3. The detailed analysis of the ARTS – Bir3 interaction provides the basis for setting it as a target for anti cancer drug design: It will enable the development of compounds that mimic ARTS CTD, remove IAPs inhibition of caspases, and thereby induce apoptosis.

## Introduction

Apoptosis, programmed cell death, is a key cellular process. Impaired apoptosis may lead to cancer [1]. Induction of cancer cell apoptosis is the essence of current anti-cancer treatments such as radiation and chemotherapy. Thus, pro- and anti-apoptotic proteins may serve as targets for anti cancer drug design based on molecular specificity.

Intrinsically disordered proteins (IDPs) or regions (IDRs) in proteins lack stable tertiary structures under physiological conditions [2], [3], [4]. IDRs usually exist in an ensemble of extended and highly flexible, dynamically interchanging conformations. These conformations may exclude or include certain elements of secondary structure giving rise to various levels of disorder [4], [5]. Such elements may be implied by the appearance of residual structure, which may serve as a basis for induced conformation upon binding [5], [6], [7]. The flexible chain and transient conformations of the IDRs are advantageous in molecular recognition, enabling high specificity and low affinity resulting in specific but easily reversed interactions [3], [8]. Various intrinsically disordered proteins are involved in numerous human diseases, including cancer [9], [10], [11]. IDPs are attractive targets for drugs that are designed to interfere with protein-protein interactions [12], [13], [14], [15].

ARTS (Apoptosis-Related protein in the TGF-beta Signaling pathway) is a pro-apoptotic mitochondrial protein and a unique member of the septin family, which functions as a tumor suppressor [16], [17]. ARTS expression is lost in more than 70% of acute lymphoblastic leukemia patients [18]. ARTS promotes apoptosis through binding and inhibiting Inhibitor of Apoptosis (IAP) proteins and specifically XIAP (X-linked IAP) [19], [20]. ARTS binding to XIAP promotes caspase activation [19]. ARTS-induced-de-repression and activation of caspases occurs through increased proteasome mediated degradation of XIAP [19], [21]. ARTS was recently shown to initiate the mitochondrial apoptotic pathway upstream of the Cytochrome C and SMAC proteins [20]. The ARTS-XIAP complex is formed immediately after induction of apoptosis, significantly before the release of SMAC and Cytochrome C from the mitochondria [20]. All IAP proteins contain at least one baculoviral IAP repeat (BIR) domain. BIR domains can directly interact with caspases and inhibit their apoptotic activity [22], [23]. XIAP is considered to be the most potent inhibitor of caspases *in vitro*, and elevated levels of this protein are found in human tumors [24], [25], [26]. ARTS specifically binds to the Bir3 domain in XIAP [20]. Deletion of the ARTS/Sept4 gene in mice promotes spontaneous development of tumors as a result of impaired apoptosis of stem and progenitor cells [27]. The surviving stem cells express increased levels of XIAP. The apoptosis, stem cell and tumor phenotypes of *Sept4*/ARTS-null mice are suppressed by inactivation of XIAP, demonstrating that ARTS acts mainly by targeting XIAP *in vivo* [27]. In addition to ARTS, mammalian XIAP antagonists have been identified, including SMAC [28], [29] and Omi/HtrA2 [30], [31]. SMAC and Omi contain a short, conserved IAP Binding Motif (IBM), used for Bir3 binding [28], [29], [32]. This motif is lacking in the ARTS sequence. The N-terminal 10 residues of SMAC, which include the IBM, did not display electron density in the unbound Smac crystal structure and were thus suggested to be disordered in the unbound state [33].

**Figure 1:**
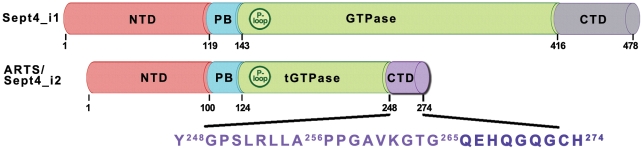
Schematic representation of ARTS domains. The domain structure of ARTS/Sept4_12 is based on its alignment with the sequence of the longer isoform Sep4_i1. The domains are: N-terminal domain, NTD, residues 1–100; polybasic linker, PB, residues 101–124; truncated GTPase domain, t-GTPase, residues 125–247; and the C-terminal domain, CTD, residues 248–274. The P-loop motif is also annotated, residues 132–139.

ARTS/Sept4_i2 is a 30.8 kDa protein of 274 residues derived by alternative splicing from the Septin 4 gene [16], [34]. Thus, ARTS shares most of its sequence with other isoforms of the Sept4 gene. Domain characterization of Sept4 [35], [36] describes three major domains including the N-terminal domain (residues 1–119), a GTPase domain (residues 144–416) and a C-terminal domain (residues 417–478) in addition to a poly-basic linker (residues 120–143). Schematic representation of ARTS domains based on its alignment with the sequence of the longest isoform Sep4_i1 is presented in Figure 1. ARTS is missing 20 N-terminal residues found in Sept4_i1, reducing the size of its N-terminal domain (NTD, residues 1–100). The subsequent 24 residues form the polybasic linker (PB, residues 101–124), similar to Sept4_i1. ARTS contains a truncated form of the GTPase domain (t-GTPase, residues 125–247) including the first 123 out of 273 residues of the original GTPase domain in spet4_i1 [35]. The remaining 27 residues in ARTS C-terminal domain (CTD, residues 248–274) constitute a unique sequence not found in any other Sept4 isoforms or other human septins. The unique C-terminus of ARTS plays an important role in the pro-apoptotic function of ARTS [19], [36]. A truncated version of ARTS lacking 68 residues from its C-terminal part (including the unique 27 residues in ARTS) lost its ability to bind XIAP [19].

Here we present a detailed quantitative analysis of the structure of ARTS CTD and its interaction with Bir3. We show that ARTS CTD is an intrinsically disordered region, which binds directly to Bir3. NMR and CD spectra of ARTS CTD showed the pattern of an IDR, in agreement with disorder predictions. Direct binding of ARTS CTD to Bir3 was demonstrated using NMR and fluorescence spectroscopy. The Bir3 interacting region in ARTS CTD was mapped to ARTS residues 266–274, which are the nine C-terminal residues in the protein. Alanine scan of ARTS 266–274 showed that His268 and Cys273 have the highest contribution to Bir3 binding. Addition of a reducing agent caused abrogation of the binding to Bir3. A dimer of ARTS 266–274, formed by oxidation of the Cys residues into a disulfide bond, bound Bir3 with a similar affinity as the monomer. We suggest that a dimer/oligomer of ARTS may be needed for the interaction with Bir3. The detailed analysis of the ARTS – Bir3 interaction provides the basis for setting it as a target for anti cancer drug design: It will enable the development of compounds that mimic ARTS CTD, remove IAPs inhibition of caspases, and thereby induce apoptosis.

## Results

### The CTD of ARTS is predicted to be disordered

**Figure 2:**
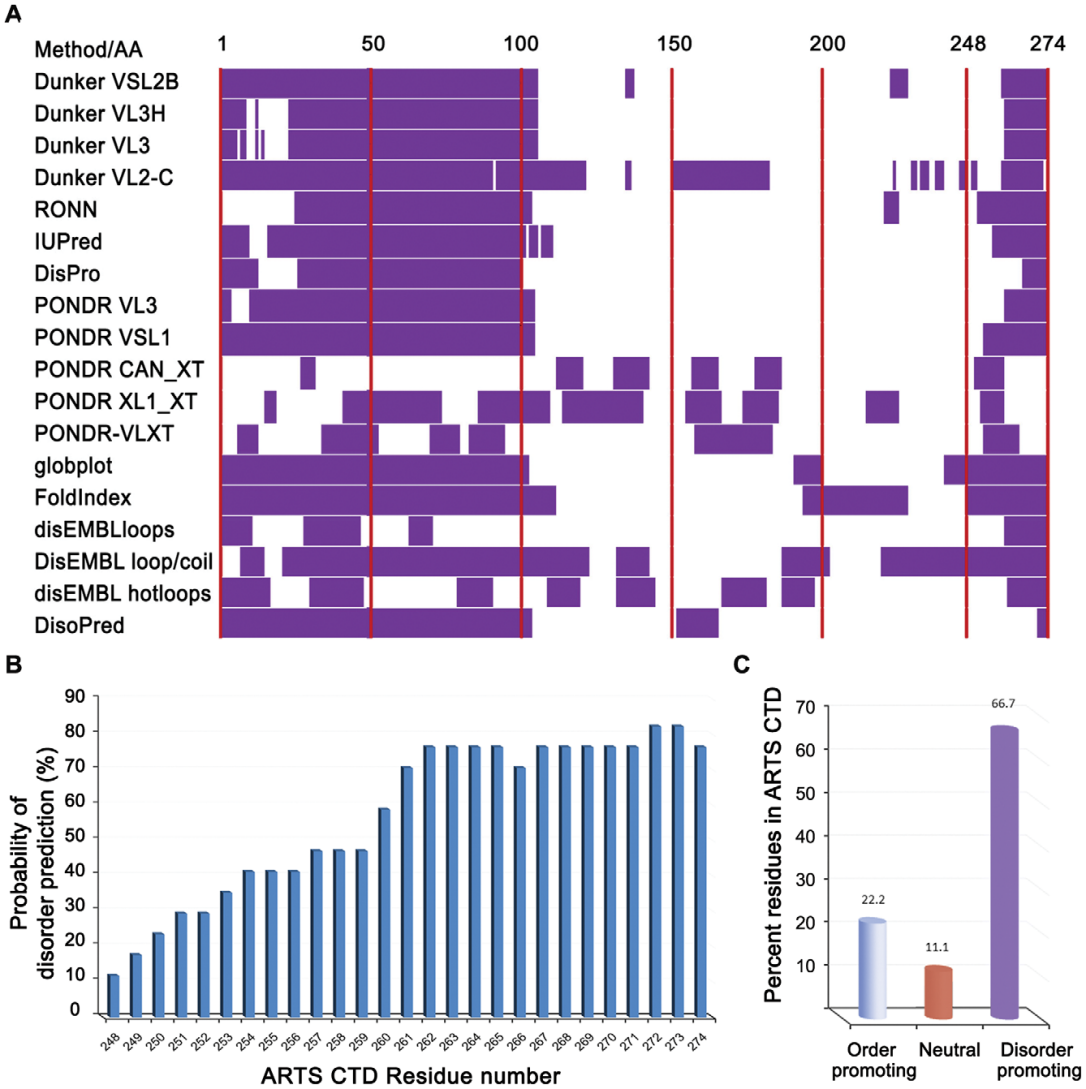
Intrinsic disorder predictions of ARTS CTD. **A:** Disorder predictions for the full length ARTS sequence were done using 18 algorithms in publicly available servers. **B:** Disorder prediction per residue in the CTD residues 248–274. Intrinsic disorder is predicted to increase towards the C-terminus. **C:** Distribution of order- and disorder-promoting residues in the sequence of ARTS CTD. The residues are classified as order promoting (I, L, V, W, F, Y, and C), disorder promoting (E, K, R, G, Q, S, P, and A) and neutral (all the others).

The sequence of the ARTS protein was submitted to 18 publicly available servers for protein disorder prediction (Figure 2A). In all cases, the full-length ARTS sequence (274 residues) was submitted and default parameters were used. Two regions were predicted to be mainly disordered: the N-terminus (residues 1–105) and the C-terminus (residues 250–274). The N-terminal domain of Sept4 (the common sequence between isoform 1 and 4) has already been suggested to be intrinsically disordered [35]. Residues 105–250 are predicted to be almost completely ordered. A statistical analysis of the residues in the CTD that are predicted to be disordered among the 18 servers is presented in Figure 2B. This presentation indicates that the major disordered region is located at the C-terminal section of the CTD, mainly between residues 260–274. The CTD sequence was analyzed for the content of order\disorder promoting residues (Figure 2C): 66.7% of the residues are disorder promoting (E, K, R, G, Q, S, P and A) and only 22.2% are order-promoting (I, L, V, W, F, Y and C). 11.1% are neutral residues.

### A peptide derived from the C-terminal domain of ARTS is intrinsically disordered: CD and NMR studies

**Figure 3:**
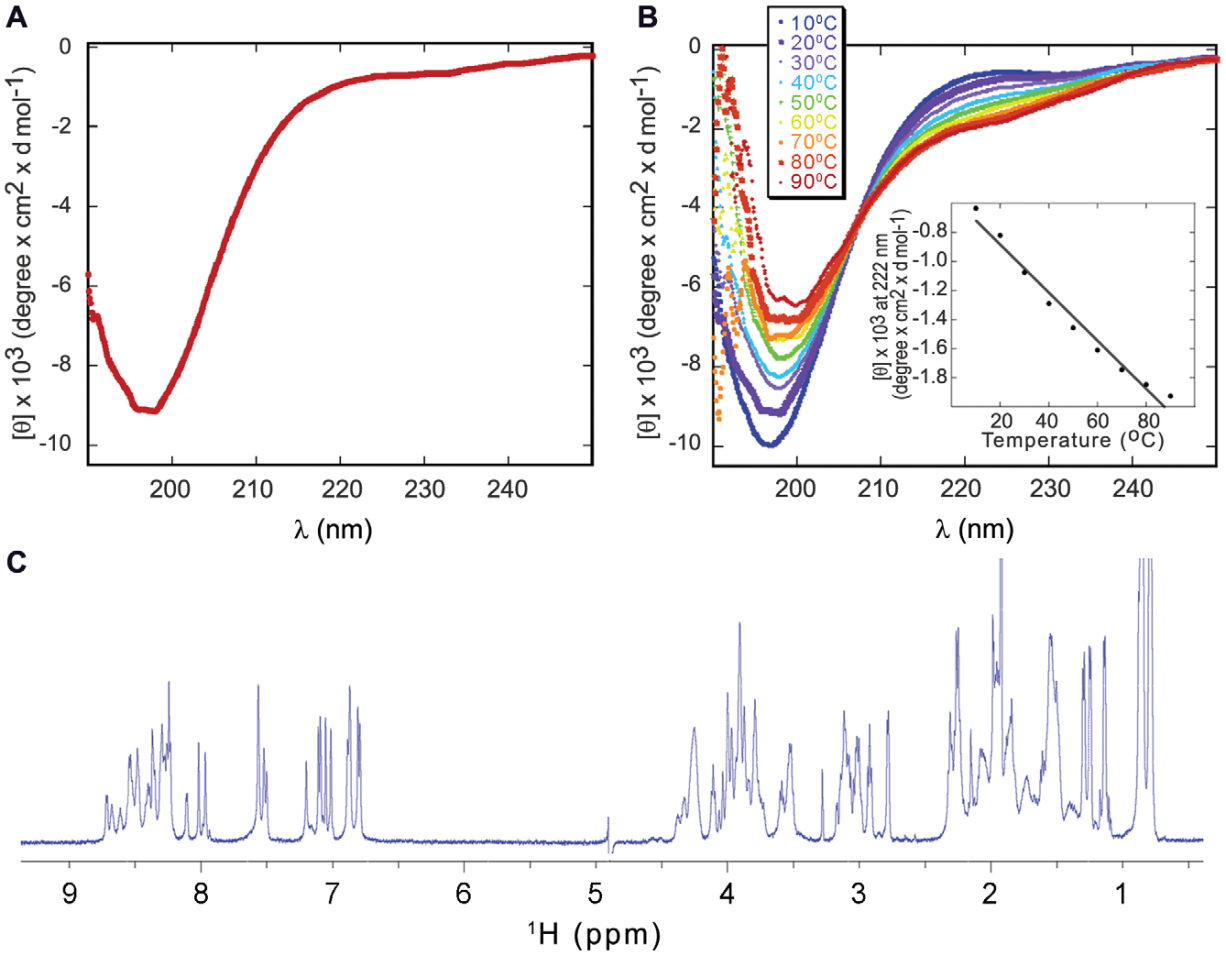
Secondary structure analysis of ARTS CTD. **A:** Far UV CD spectrum of ARTS CTD in phosphate buffer, pH 7.4 at 20°C and an ionic strength of 50 mM. The strong absorption at 200 nm indicates a pattern of unfolded region, where α-helices or β-strands characteristic are absent. **B:** The effect of temperature on the CD spectrum of ARTS CTD. All experiments were performed in 20 mM phosphate buffer, pH 7.4 at 20°C and an ionic strength of 50 mM. Peptide concentration was 60 µM. The temperature range was 10–90°C. A linear increase of the CD signal at 222 nm as a function of temperature is consistent with an apparent temperature-induced formation of a residual structure. **C:** The ^1^H-NMR spectrum of ARTS CTD in 90% 20 mM deuterated Hepes buffer and10% D_2_O, pH 6.8, at 15°C, ionic strength of 50 mM. The resonances have a narrow dispersion, as expected for an unstructured polypeptide. The fingerprint resonances range between 8.75–7.95 ppm, Δ = 0.80 ppm.

The structural properties of the ARTS C-terminal domain (CTD, residues 248–274) were studied using CD and NMR spectroscopy. The far-UV CD spectrum of ARTS CTD in 20 mM phosphate buffer, pH 7.4, at 20°C (Figure 3A) is composed largely of a minimum at 200 nm and is missing the typical signatures of secondary structures, indicating mainly a random coil. This is a characteristic pattern for disordered regions. Calculating the secondary structure content of ARTS CTD using the DichroWeb server [37], [38] indicated a high content of disordered regions corresponding to 48% of the total secondary structured elements (Helix 2%, Strand 31%, Turns 17%, data not shown). Changes of the signal at 222 nm as a function of temperature from 10°C to 90°C showed a linear increase of ellipticity with increasing temperature (Figure 3B). This indicates an apparent temperature-induced formation of residual secondary structure. This set of CD spectra also revealed a well-defined isodichroic point at 207 nm, which may represent equilibrium among the unordered conformations and the induced conformation. Calculation of the changes in secondary structure content as a function of the temperature using DichroWeb showed that the fraction of the disordered structures decreased upon increasing the temperature from 49% at 10°C to 33% at 50°C. The ^1^H-NMR spectra of ARTS CTD showed a narrow dispersion of amide resonances, ranging from 8.75–7.95 ppm, Δ = 0.80 ppm. This pattern implies a random coil conformation and is characteristic of disordered regions and peptides, where residues are not held in a defined structure [39] (Figure 3C).

### Quantification of the interaction between the ARTS CTD and Bir3

**Figure 4:**
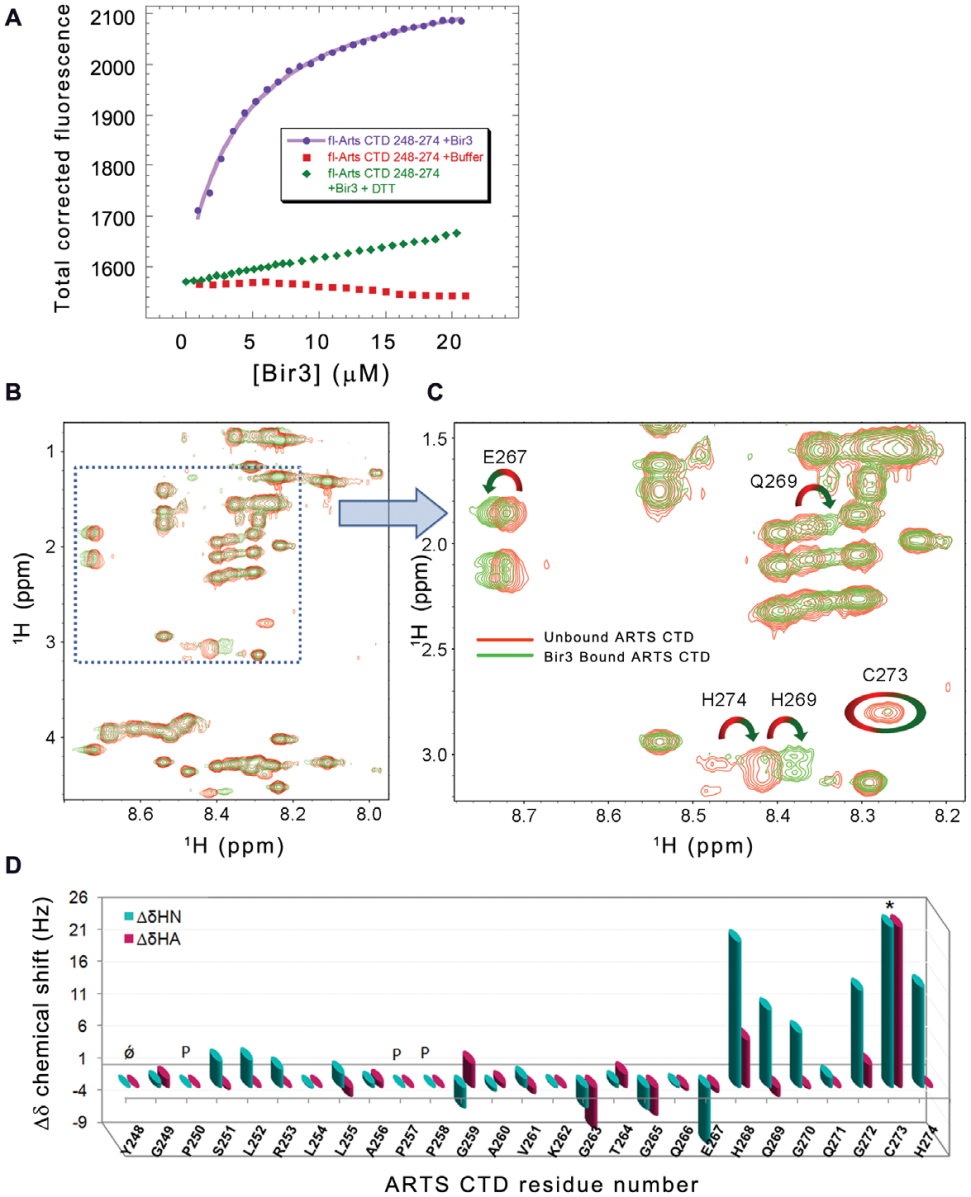
Quantitative analysis of the interaction between ARTS CTD and Bir3. **A:** Binding of ARTS CTD to Bir3. Bir3 was titrated into fluorescein- labeled ARTS CTD 248–274 (purple) resulting in *K*_d_ = 8.0±0.7 µM. Titration of Bir3 in the presence of 2 mM DTT resulted in no significant binding (green). **B** and **C:** NMR TOCSY spectra of free ARTS CTD (red) and bir3 – bound ARTS CTD (green) show backbone amide chemical shift deviations upon binding. **B:** Finger print resonances and **C:** enlargement. **D:** NMR mapping of the ARTS residues that mediate its binding to Bir3. Shown are the chemical shift changes of ARTS CTD upon interaction with Bir3. The interacting ARTS residues are within the nine C-terminal residues. The largest changes are displayed by residues E267, H268, Q269, C273 and H274.

Fluorescence anisotropy was used to characterize the interaction between ARTS CTD and the Bir3 subunit of XIAP. ARTS CTD (residues 248–274) was synthesized using standard Fmoc chemistry and labeled with fluorescein at its N-terminus. The Bir3 subunit of the XIAP (residues 252–350) was expressed with a His-tag at the C-terminus and purified (see materials and methods and Figure S1). The binding experiments were done in 20 mM Hepes buffer, pH 7.4, 45 mM NaCl, at 10°C. Bir3 was titrated into fl-ARTS CTD and the data were fit to 1∶1 binding model resulting in *K*_d_ = 8.0±0.7 µM (Figure 4A). Addition of a reducing agent (2 mM DTT) severely impaired the interaction between ARTS CTD and Bir3 (Figure 4A).

### Mapping of the ARTS CTD residues that mediate its interaction with Bir3

NMR analysis of the Bir3-bound versus unbound ARTS CTD was used to reveal the ARTS residues that mediate its interaction with Bir3, which itself was too large to be seen in the spectra. The TOCSY spectra were assigned using the NOESY spectra and then compared. Figure 4B and C shows the overlay of the TOCSY spectra of the free (red) and Bir3-bound (green) ARTS CTD. The deviations of backbone Hα and HN proton chemical shifts upon Bir3 binding are shown in Figure 4D. The overall dispersion of amide resonances in the Bir3-bound state remained narrow, indicating that there was no major structural change in the overall conformation of ARTS CTD upon Bir3 binding. Changes in the chemical shifts of specific residues in ARTS CTD indicate a change in their chemical environment in the presence of Bir3. This indicates the involvement of these residues in the interaction. The residues in the C-terminal part of ARTS CTD underwent the largest changes in chemical shift upon binding Bir3. His268 and Cys 273 showed the largest chemical shift changes: The HIS268 peak showed a significant chemical shift change while the Cys273 peak almost completely disappeared in the presence of Bir3. An alternative Cys peak corresponding to the newly bound conformation was absent. The intensity of the His274 peak also decreased, though to a lesser extent. In this case, a different peak was present in the bound state. The His274 peak as well as the peaks of E267, Q269, G270 and G272 also showed significant chemical shift changes upon binding Bir3. Residues 248–266 did not show significant chemical shift changes upon binding Bir3, indicating they are not involved in the interaction. Thus, the Bir3 binding region in ARTS CTD was mapped to the nine C-terminal residues of ARTS.

**Figure 5:**
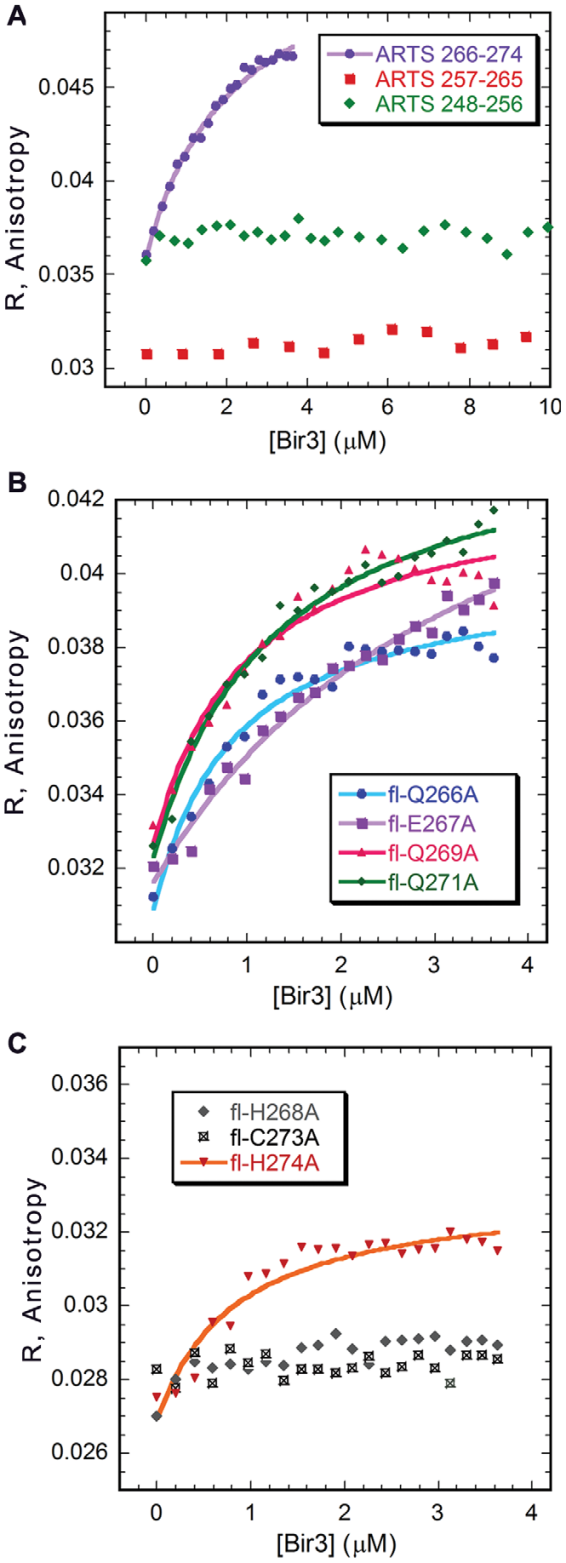
Alanine scan of ARTS CTD. **A:** Fluorescence anisotropy binding studies of Bir3 and peptides derived from ARTS CTD: only ARTS 266–274 bound Bir3, with a *K*_d_ of 2.9±0.3 µM, while ARTS 248–256 and ARTS 257–265 did not. **B** and **C:** Alanine scan of ARTS CTD 266–274: binding studies using fluorescence anisotropy. Substituting H268 and C273 by alanine impaired the binding of ARTS CTD to Bir3, while alanine substitutions of Q266, E267, Q269 and Q271 mutations to Bir3 did not affect binding. Binding affinities are given in Table 1.

Based on the NMR results, we divided ARTS CTD 248–274 into three non-overlapping peptides that covered its entire sequence: ARTS 248–256, ARTS 257–265 and ARTS 266–274 (Table 1). The peptides were synthesized and labeled with fluorescein and their binding to Bir3 was studied by fluorescence anisotropy. Only fl-ARTS 266–274, derived from the nine C-terminal residues of the protein, bound Bir3 with *K*_d_ = 2.5±0.3 µM. The peptides derived from ARTS 248–265 did not show any binding (Figure 5A), in agreement with the NMR results.

**Table 1:** ARTS CTD derived peptides: sequences and binding affinity to Bir3.

Peptide name	Sequence	*K*_d_ (µM)
fl-ARTS 248–274	YGPSLRLLAPPGAVKGTGQEHQGQGCH	8.0±0.7
fl-ARTS 248–256	YGPSLRLLA	nb
fl-ARTS 257–265	PPGAVKGTG	nb
fl-ARTS 266–274	QEHQGQGCH	2.5±0.3
Dimer fl-ARTS 266–274	QEHQGQGCH(S-S)HCGQGQHEQ	2.40±0.02
fl-ARTS 266–274 Q266A	**A**EHQGQGCH	0.9±0.1
fl-ARTS 266–274 E267A	Q**A**HQGQGCH	3.6±0.8
fl-ARTS 266–274 H268A	QE**A**QGQGCH	nb
fl-ARTS 266–274 Q269A	QEH**A**GQGCH	1.0±0.3
fl-ARTS 266–274 Q271A	QEHQG**A**GCH	1.3±0.2
fl-ARTS 266–274 C273A	QEHQGQG**A**H	nb
fl-ARTS 266–274 H274A	QEHQGQGC**A**	0.8±0.3

nb, no binding detected. Alanine substitutions are indicated in bold.

To verify the NMR results and assess the contribution of each ARTS residue to Bir3 binding we performed an alanine scan of ARTS 266–274. A series of fluorescein labeled peptides were synthesized based on the sequence of ARTS 266–274. In each peptide one residue was replaced by alanine. The binding affinity of the alanine-substituted peptides to Bir3 was determined by fluorescence anisotropy (Figure 5B and C, Table 1). ARTS 266–274 H268A and Cys273A did not bind Bir3. The alanine scan results are in agreement with the NMR results, corroborating the role of His268 and Cys273 in the interaction with Bir3.

### ARTS CTD needs to be dimeric to bind Bir3

**Figure 6:**
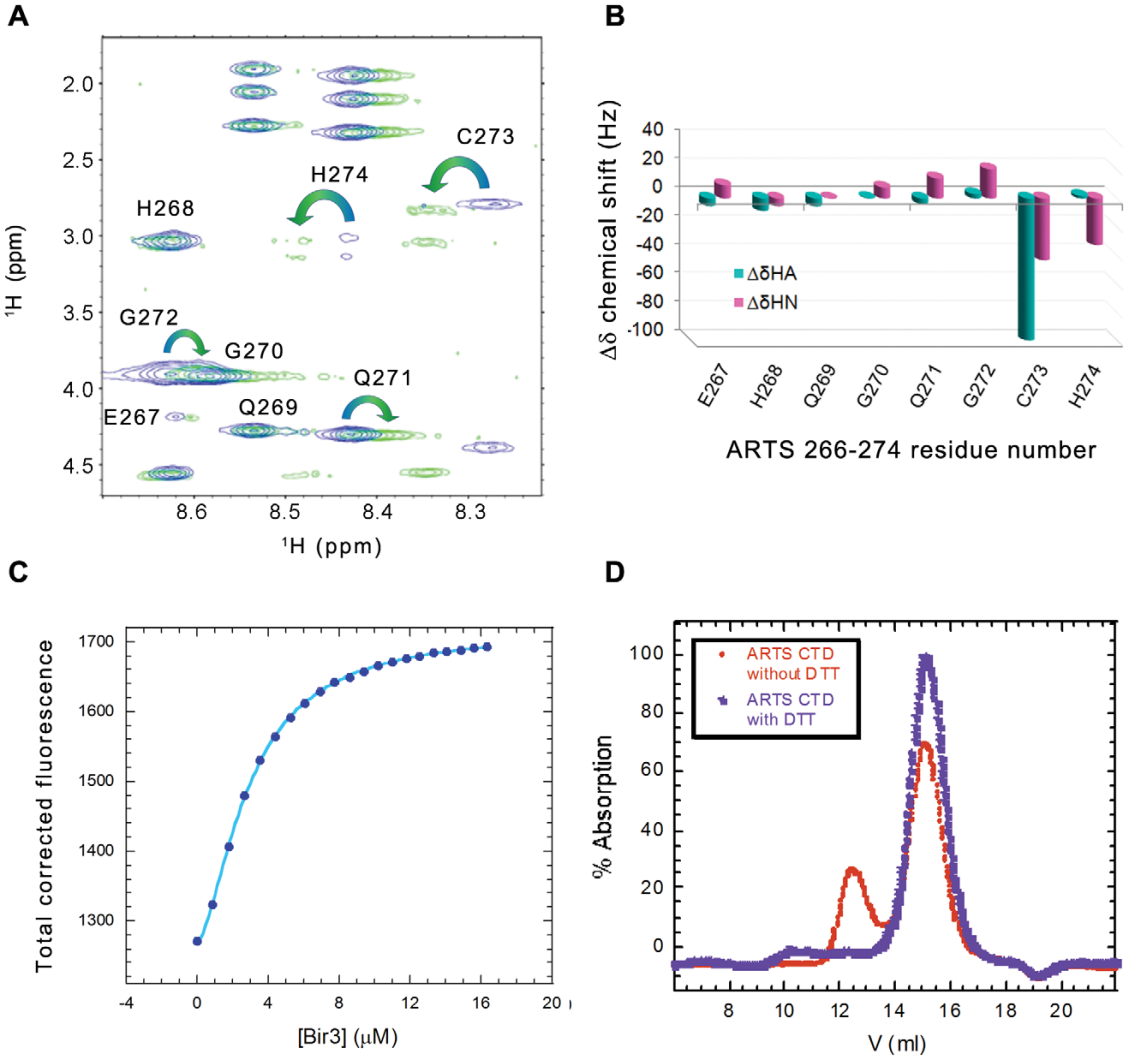
Dimer formation by the ARTS CTD peptide. **A** and **B:** NMR analysis of the oxidized versus non-oxidized ARTS 266–274. Overlay of the TOCSY spectra of the fingerprint region (**A**) showing splitting of the Hβ Cys273 into two signals, coupled with deviation of the chemical shift (**B**) of the neighboring residues H274, G 272 and Q271. **C:** Binding of the dimeric fl-ARTS 266–274 to Bir3 using fluorescence spectroscopy. **D:** Analytical size exclusion chromatography of ARTS CTD with (Blue) and without (red) 2 mM DTT. In the absence of a reducing agent a second peak of ARTS dimer was present.

The importance of Cys273 residue for Bir3 binding raised the question of whether disulfide bond formation is involved in the interaction with Bir3. Addition of a reducing agent weakened the interaction between the ARTS derived peptides and Bir3 (Figure 4A). This raised the possibility that a disulfide bond is formed between two ARTS peptide molecules to form an active dimer. To test this, a covalent dimer of ARTS 266–274 was prepared by forming a disulfide bond between the Cys273 residues of two peptide monomers. MS gave a single peak corresponding the mass of the dimer (data not shown). Formation of the disulfide bond was further supported by NMR, where a single set of peaks was observed in which the Hα proton peaks of Cys273 were split into two different peaks, representing two different orientations of the hydrogens on the Cys273 Cβ carbon (Figure 6A, B). Changes in the chemical shift of the sequentially following His274 and preceding Gln272 were also observed. Binding of Bir3 to the dimeric fl-ARTS 266–274 was studied using fluorescence spectroscopy (Fig. 6C). The results were fit to the Hill equation resulting in *K*_d_ = 2.4±0.1 µM and a Hill coefficient of 1.5±0.1. These results suggest that one dimer of ARTS266–274 is capable of binding at least two molecules of Bir3 in a cooperative manner and indicates binding of a mixed monomer-dimer population to Bir3. The apparent averaged *K*_d_ deduced from the best fit to Hill model is similar to ARTS 266–274 monomer binding to Bir3. These results imply that ARTS CTD may bind Bir3 as a dimer and Cys 273 may be important for the dimerization of the CTD and not necessarily for direct binding to Bir3.

To confirm its oligomeric state, ARTS CTD 248–274 was analyzed by analytical gel filtration. The identities of all the peaks were verified by mass spectrometry (Figure S2). In the presence of 2 mM DTT, ARTS CTD eluted from the gel filtration column as a single peak that corresponded to the mass of the monomer. In the absence of DTT an additional peak of mixed dimer and monomer eluted earlier.

## Discussion

Using structural and biophysical methods, we showed that the nine C-terminal residues (266–274) of the proapoptotic ARTS protein mediate its interaction with the Bir3 domain of XIAP. These residues are in the unique C-terminal domain of ARTS. The far-UV CD and NMR spectra of ARTS CTD indicated that the CTD is disordered and lacks significant secondary structure elements. The increase of the CD signal at 222 nm with increasing temperature, as well as the isodichroic point, indicate a conformational equilibrium between two states. This pattern implies the possible existence of a temperature induced residual structure, a common property of IDPs [7], [40]. The residual structure may serve as a starting point for an induced specific conformation of the CTD upon interacting with binding partners such as Bir3. The Bir3 binding residues in SMAC are also located in a disordered terminal region. This may indicate a role for terminal disordered residues in Bir3 inhibition.

Detailed mapping of the ARTS residues that mediate its binding to Bir3 showed that His268 and Cys273 have the largest contribution to the interaction. His268 showed the largest deviation of NMR chemical shift upon Bir3 binding, while the NMR peak of Cys273 almost completely disappeared in the presence of Bir3. Mutating these residues to alanine abolished the interaction with Bir3. Cys273 may have a role in ARTS CTD dimerization and not only in direct binding to Bir3. This was demonstrated by inhibition of the ARTS CTD-Bir3 interaction by a reducing agent. Furthermore, the disulfide dimeric ARTS 266–274, in which the disulfide bond was formed specifically between the Cys273 residues of the two monomers, bound tightly to Bir3 with a Hill coefficient of 1.5, indicating binding of a mixed monomer-dimer population to Bir3. This indicates that each ARTS CTD monomer is able to bind one Bir3 subunit. Dimer formation may also explain the disappearance of the Cys273 peak from the NMR TOCSY spectrum upon addition of Bir3. Formation of a disulfide bond between the Cys273 residues may impose geometric strain on this region, enabling increased exchange of amide protons that results in disappearance of the signal. The single set of peaks, together with the high NMR sample concentration, suggests that the predominant form was dimeric. We conclude that dimer formation of ARTS CTD may be important for its interaction with Bir3.

XIAP and other proteins from the IAP family are attractive targets for anti-cancer drug design due to their elevated expression in cancer cells and their association to chemo-resistance [25], [41]. XIAP also contributes to metastasis formation *in vivo* [42]. The most common small molecule IAP antagonists are mimetics of endogenous IAP inhibitors such as SMAC/DIABLO, and some of them are currently undergoing phase I and II clinical trials [41], [43]. ARTS was shown to function as a tumor suppressor protein both in human and mice studies [18], [27]. Furthermore, the apoptosis, stem cell and tumor phenotypes of *Sept4*/ARTS-null mice were suppressed by inactivation of XIAP, demonstrating that ARTS acts mainly by targeting XIAP *in vivo* [27]. Understanding the ARTS – Bir3 interaction at the molecular level is essential for developing peptides and small molecules based on the ARTS CTD that would be able to inhibit XIAP. Future studies are required to determine the ARTS binding sites on Bir3. Our results lay the molecular basis for designing a new set of anti-IAPs lead compounds that mimic the ARTS function, inhibit IAPs and promote apoptosis of cancer cells.

## Materials and Methods

### Peptide synthesis, labeling and purification

ARTS derived peptides were synthesized using standard SPPS methods on a Liberty Microwave-Assisted Peptide Synthesizer (CEM) using standard Fmoc (9-fluorenylmethoxycarbonyl) chemistry as described [44]. The peptides were labeled using 5′ and 6′ carboxyfluorescein succinimidyl ester (Molecular Probes, Carlsbad, CA) at their N terminus, as described in [45]. The peptides were purified on a Gilson high-pressure liquid chromatography using a reverse-phase C8 semi-preparative column (ACE) with varying gradients of acetonitrile in water (both containing 0.001% (v/v) trifluoroacetic acid). The peptides were analyzed by mass spectrometry (Voyager DE-PRO, applied biosystems). Oxidation of the labeled and unlabeled ARTS 266–274 was carried out manually in solution after HPLC purification of the parent peptide. The peptide (∼0.5 mg/ml) was dissolved in DMSO and diluted to 20% DMSO in 5% aqueous acetic acid. The pH was adjusted to 6. The reaction was performed overnight with mild shaking [46]. The peptides were purified again after the oxidation reaction.

### BIR3 expression and purification

The pET-21b(+) vector containing XIAP (252–350) Bir3 domain with a C-terminal His Tag was transformed into E. coli strain BL21 Star^+^ (DE3) (Novagen). Cells were grown at 37°C in LB medium to an optical density (600 nm) of ∼0.6 and induced with 0.4 mM isopropyl-β-D-1-thiogalactoside (IPTG). Cells were harvested after 8 h of incubation at 37°C and were lysed by Microfluidizer (Microfluidics). The protein was purified on HiTrap nickel-Sepharose FF column (100×10 mm, GE Healthcare), using an FPLC system (ÁKTA explorer, Amersham Biosciences). The eluted protein was further purified by filtration on a Sephacryl S100 column (950×26 mm; Amersham Biosciences). The protein was stored in 20 mM Hepes pH 7.4, 50 mM NaCl, 10 mM βME, 0.02% sodium azide and was dialyzed before experimental use.

### Circular dichroism

CD spectra were recorded using a J-810 spectropolarimeter (Jasco) in 25 mM potassium phosphate buffer, pH 7.3, 120 mM KCl and 20 µM ARTS CTD 248–274, in a 0.1 cm quartz cuvette for far-UV CD spectroscopy. Far-UV CD spectra were collected over a spectral range of 185 nm to 260 nm. Data was collected each 1 nm and averaged over 5 acquisitions. Prior to each experiment the peptide was centrifuged at 13,200 rpm for 5 min. Wavelength scans were corrected for buffer contributions and converted to molar ellipticity. Changes in the CD spectra were monitored as a function of temperature from 10°C to 90°C with 10°C steps, as described above. The molar ellipticity at 222 nm was fit to a linear model. The secondary structure content was calculated using DichroWeb [37], [38] with the CDSSTR analysis algorithm.

### Analytical gel filtration

Analytical gel filtration of purified ARTS CTD 248–274 was performed on an ÄKTA Explorer (GE Healthcare- Amersham Pharmacia, Giles, U.K.) using a Superose 30 analytical column 30×1 cm (GE Healthcare) equilibrated with 20 mM Hepes buffer, pH 7.4 and 50 mM NaCl. ARTS CTD concentrations of, 1–2 mM were tested. ARTS CTD eluted with a flow rate of 1 ml/min at 4°C and the elution profile was recorded by continuously monitoring the UV absorbance at 220 nm. The eluted peaks were analyzed for their molecular weight using mass spectrometry.

### NMR

ARTS CTD derived peptides in lyophilized form were dissolved in an aqueous solution (0.5–1.5 mM) of 20 mM deuterated -Hepes buffer, 45 mM potassium chloride (Aldrich) and 0.02% (w/v) sodium azide. Bir3 was dialyzed overnight against the deuterated Hepes buffer prior to the experiment. The apparent pH was adjusted to 6.8±0.2 with NaOH and the sample was centrifuged. The NMR experiments were performed at 12±0.1°C, which was found to be the optimal temperature for maximal conformational stability without broadening. The instrument was a Bruker Avance 600 MHz DMX spectrometer, with a 5-mm selective probe equipped with a self-shielded xyz-gradient coil. TOCSY [47], COSY [48] and NOESY [49] spectra measured under identical experimental conditions were used for resonance assignment, using the sequential assignment methodology [50]. Spectra were processed and analyzed with the TopSpin (Bruker Analytische Messtechnik GmbH) and SPARKY (T. D. Goddard and D. G. Kneller, SPARKY 3; University of California, San Francisco http://www.cgl.ucsf.edu/home/sparky/). Chemical shifts deviations of ARTS CTD in the Bir3 bound and unbound were calculated from the TOCSY spectra using SPARKY.

### Binding studies using fluorescence spectroscopy

Measurements were performed at 10°C by using a PerkinElmer LS-50b luminescence spectrofluorimeter equipped with a Hamilton microlabM dispenser as described in [44]. The fluorescently labeled peptide concentrations were determined by using a UV spectrophotometer (Shimadzu, Kyoto, Japan). The fluorescein-labeled ARTS derived peptides were dissolved in 20 mM Hepes buffer, pH 7.4 and 45 mM NaCl. 1 ml of the labeled peptide solution was placed in a cuvette, and the non-labeled Bir3 (200 µl, 150 µM) was placed in the dispenser. Additions of 5 µl were titrated at 90 sec intervals, the solution was stirred for 15 sec, and the fluorescence and anisotropy were measured after each addition by using an excitation wavelength of 480 nm and an emission wavelength of 530 nm. Dissociation constants (*K*_d_) were calculated by fitting the fluorescence titration curves (corrected for dilution) or fluorescence anisotropy curves to 1∶1 binding model using KALEIDAGRAPH (Synergy Software, Reading, PA). The following equation was used for the single-site model of the fluorescence results:
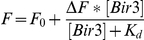
Where, F_0_ is the initial fluorescence, ΔF is the amplitude of the fluorescence change from unbound and bound states, *K*_d_ is the dissociation constant and [Bir3] is the added concentration of Bir3 domain. A similar equation was used to fit the anisotropy results by replacing the fluorescence (F) with anisotropy (R) changes.

The Hill equation was used for fitting the binding of Bir3 to the dimeric fl-ARTS 266–274 peptide:
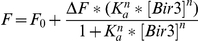
Where, F_0_ is the initial fluorescence, ΔF is the amplitude of the fluorescence change from unbound and bound states, *K*_a_ is the association constant, [Bir3] is the added concentration of Bir3 and *n* is the Hill coefficient.

### Disorder predictions

The sequence of the full length ARTS (GenBank code: AAG45673.1) was submitted to 10 publicly available servers implementing 18 different algorithms for protein disorder prediction. In all cases we used the default parameters. The methods are reviewed in Ferron et al. [51].

## Supporting Information

Figure S1 **Bir3 expression and purification.** The expressed Bir3 domain of XIAP (252–350) was purified in two steps: **A.** Nickel affinity chromatography. Absorbance at 280 nm is shown in red and the absorbance at 260 nm in blue. Addition of the elution buffer containing 250 mM Imidazole is shown in green line. Fraction numbers are indicated in red in the bottom of the graph. Bir3 eluted around 30% elution buffer. **B:** SDS-PAGE of the fractions indicates the presence of Bir3 in fractions 10–20. Fractions 11–19 were collected for gel filtration purification. **C:** Gel filtration chromatogram of Bir3 Absorbance at 280 nm is shown in red and the absorbance at 260 nm in blue.. Bir3 eluted in the main peak (fractions 7–11). **D:** SDS-PAGE of the main peak fractions indicating the high purity of Bir3.
(TIF)

Figure S2 **Mass Spectrum analysis of the analytical size exclusion chromatography results.** The mass spectrum of two representative fractions from each of the peaks eluted from the analytical gel filtration experiments: A. In the absence of a reducing agent two different peaks eluted. The early fraction included the mass of ARTS CTD monomer as well as dimer whereas the later peak includes practically only the monomer. B. In the presence of 2 mM DTT only one peak corresponding to the monomer eluted from the column.
(TIF)
