# Supplementary figures and images for: Mechanism of the Interaction between the Intrinsically Disordered C-Terminus of the Pro-Apoptotic ARTS Protein and the Bir3 Domain of XIAP
Source: PLoS One. 2011 Sep 20;6(9):e24655. doi: 10.1371/journal.pone.0024655 (PMC3176765; doi:10.1371/journal.pone.0024655)

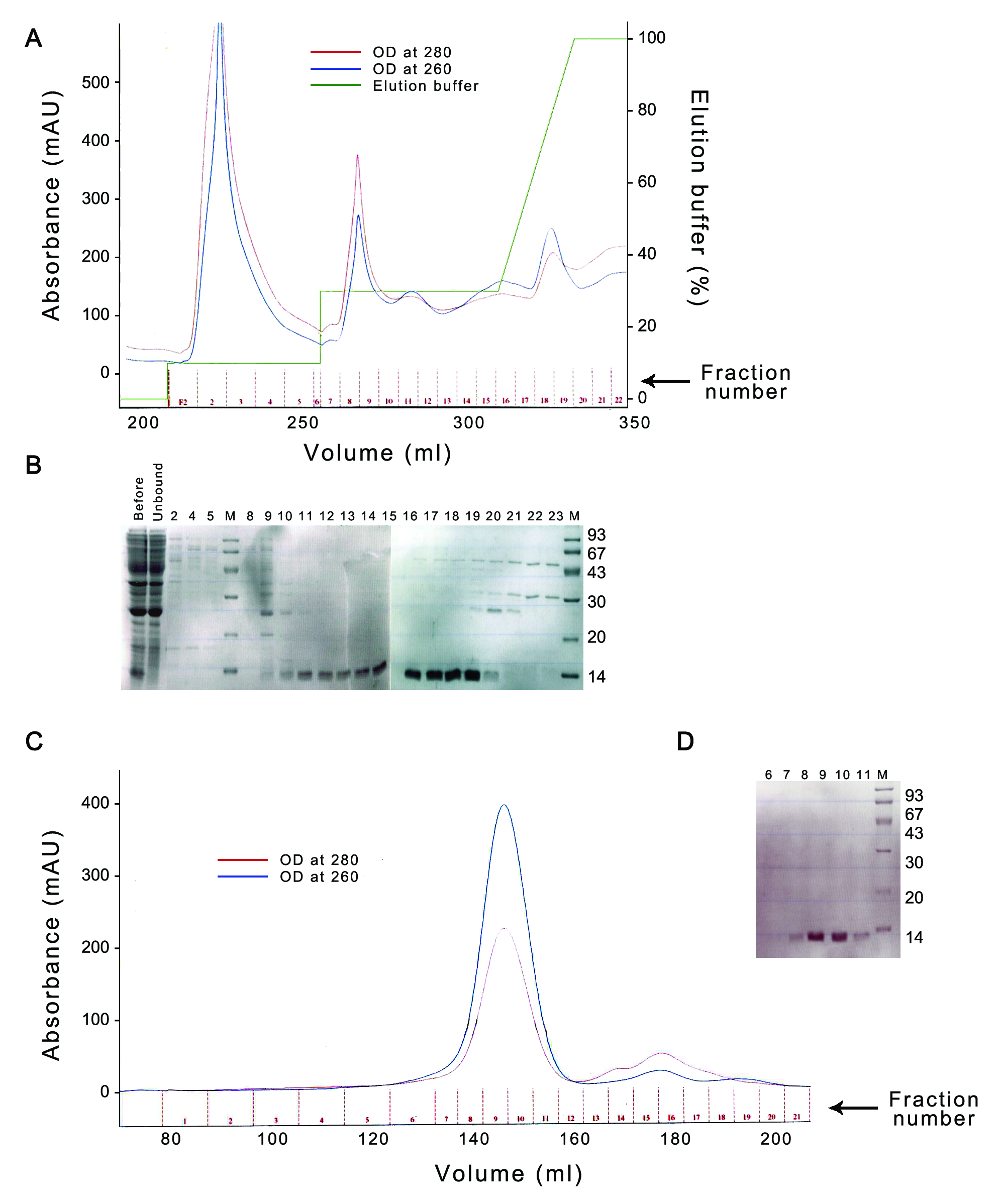

Supplement: Figure S1 — Bir3 expression and purification. The expressed Bir3 domain of XIAP (252–350) was purified in two steps: A. Nickel affinity chromatography. Absorbance at 280 nm is shown in red and the absorbance at 260 nm in blue. Addition of the elution buffer containing 250 mM Imidazole is shown in green line. Fraction numbers are indicated in red in the bottom of the graph. Bir3 eluted around 30% elution buffer. B: SDS-PAGE of the fractions indicates the presence of Bir3 in fractions 10–20. Fractions 11–19 were collected for gel filtration purification. C: Gel filtration chromatogram of Bir3 Absorbance at 280 nm is shown in red and the absorbance at 260 nm in blue.. Bir3 eluted in the main peak (fractions 7–11). D: SDS-PAGE of the main peak fractions indicating the high purity of Bir3. (TIF) [file pone.0024655.s001.tif]

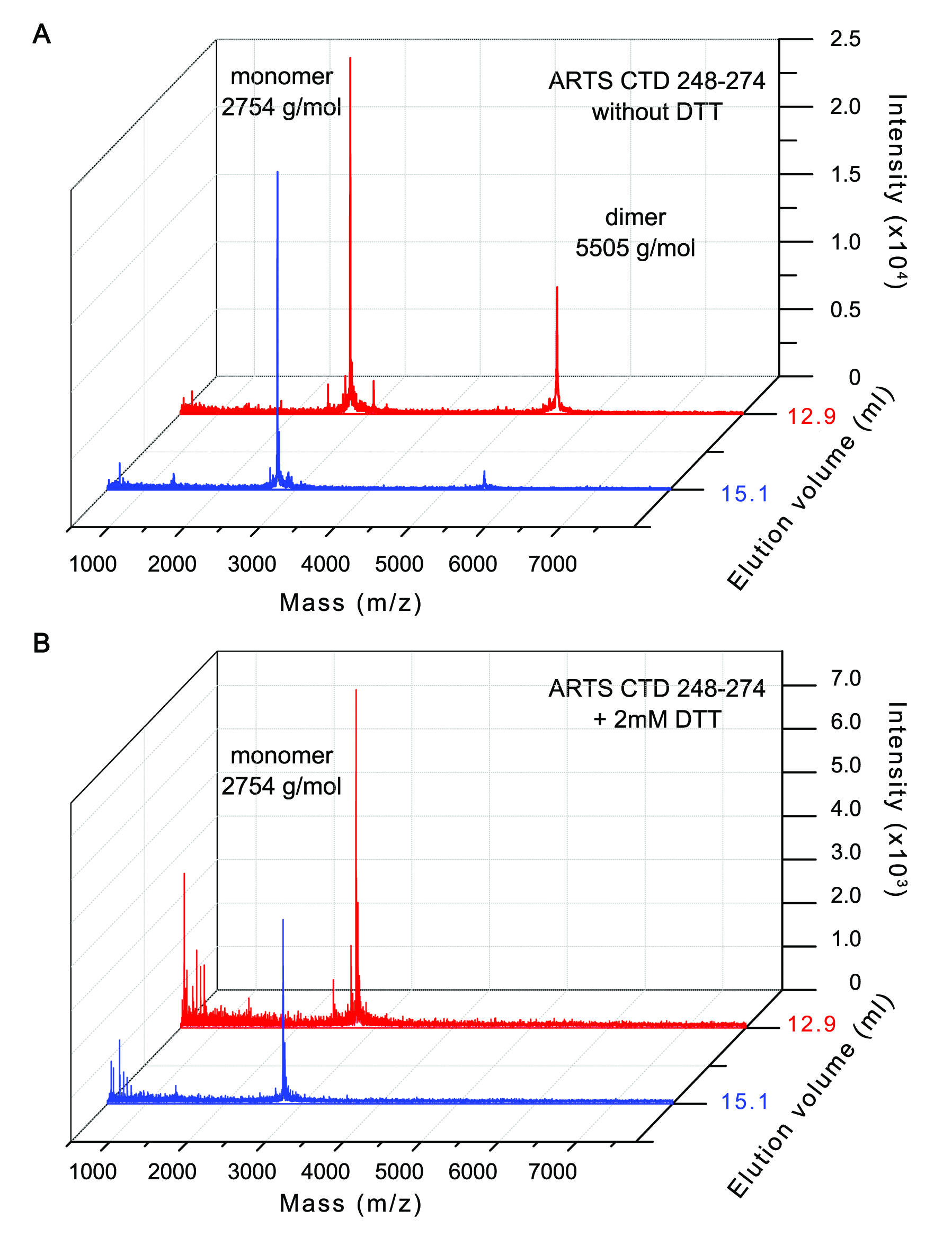

Supplement: Figure S2 — Mass Spectrum analysis of the analytical size exclusion chromatography results. The mass spectrum of two representative fractions from each of the peaks eluted from the analytical gel filtration experiments: A. In the absence of a reducing agent two different peaks eluted. The early fraction included the mass of ARTS CTD monomer as well as dimer whereas the later peak includes practically only the monomer. B. In the presence of 2 mM DTT only one peak corresponding to the monomer eluted from the column. (TIF) [file pone.0024655.s002.tif]
